# Addressing the “Black Hole” of Low Back Pain Care With Clinical Decision Support: User-Centered Design and Initial Usability Study

**DOI:** 10.2196/66666

**Published:** 2025-02-04

**Authors:** Robert S Rudin, Patricia M Herman, Robert Vining

**Affiliations:** ^1^RAND, 20 Park Plaza, Suite 910, Boston, MA, 02116, United States, 1 6173382059 ext 8636, 1 6173577470; 2 RAND, Santa Monica, CA, United States; 3 Palmer Center for Chiropractic Research, Palmer College of Chiropractic, Davenport, IA, United States

**Keywords:** low back pain, clinical decision support, user-centered design, usability, back pain, low back pain care, pain, clinical decision, societal burden, substantial, burden, evidence-based, treatment, diagnosis, support tool, clinicians, chiropractic, chiropractor, reviews, scenario-based interviews, interviews

## Abstract

**Background:**

Low back pain (LBP) is a highly prevalent problem causing substantial personal and societal burden. Although there are specific types of LBP, each with evidence-based treatment recommendations, most patients receive a nonspecific diagnosis that does not facilitate evidence-based and individualized care.

**Objectives:**

We designed, developed, and initially tested the usability of a LBP diagnosis and treatment decision support tool based on the available evidence for use by clinicians who treat LBP, with an initial focus on chiropractic care.

**Methods:**

Our 3-step user-centered design approach consisted of identifying clinical requirements through the analysis of evidence reviews, iteratively identifying task-based user requirements and developing a working web-based prototype, and evaluating usability through scenario-based interviews and the System Usability Scale.

**Results:**

The 5 participating users had an average of 18.5 years of practicing chiropractic medicine. Clinical requirements included 44 patient interview and examination items. Of these, 13 interview items were enabled for all patients and 13 were enabled conditional on other input items. One examination item was enabled for all patients and 16 were enabled conditional on other items. One item was a synthesis of interview and examination items. These items provided evidence of 12 possible working diagnoses of which 3 were macrodiagnoses and 9 were microdiagnoses. Each diagnosis had relevant treatment recommendations and corresponding patient educational materials. User requirements focused on tasks related to inputting data, and reviewing and selecting working diagnoses, treatments, and patient education. User input led to key refinements in the design, such as organizing the input questions by microdiagnosis, adding a patient summary screen that persists during data input and when reviewing output, adding more information buttons and graphics to input questions, and providing traceability by highlighting the input items used by the clinical logic to suggest a working diagnosis. Users believed that it would be important to have the tool accessible from within an electronic health record for adoption within their workflows. The System Usability Scale score for the prototype was 84.75 (range: 67.5‐95), considered as the top 10th percentile. Users believed that the tool was easy to use although it would require training and practice on the clinical content to use it effectively. With such training and practice, users believed that it would improve care and shed light on the “black hole” of LBP diagnosis and treatment.

**Conclusions:**

Our systematic process of defining clinical requirements and eliciting user requirements to inform a clinician-facing decision support tool produced a prototype application that was viewed positively and with enthusiasm by clinical users. With further planned development, this tool has the potential to guide clinical evaluation, inform more specific diagnosis, and encourage patient education and individualized treatment planning for patients with LBP through the application of evidence at the point of care.

## Introduction

Low back pain (LBP) is highly prevalent globally, causing personal and societal burden, chronic disability, and substantial health care expenditures, ranging from US $62 to US $124 billion annually in the United States [1-6]. Most LBP is caused by 1 or more benign conditions that are not associated with dangerous pathology [7]. Instead, LBP is influenced by injury, inflammation, and dysfunction of myofascial tissues, joints, and nerve roots or peripheral nerves, with symptoms also influenced by nonphysical factors (eg, psychological, social, and environmental) and central sensitization processes [8]. However, despite extensive evidence in the literature informing diagnoses and treatments for specific types of LBP, nonpathological LBP is typically referred to as nonspecific [7]. The nonspecific LBP label lacks details to offer a working understanding of a patient’s condition or inform personalized treatment plans. Furthermore, some diagnoses for common “specific” and neurologically related conditions, such as stenosis, neurogenic claudication, radiculopathy, and sciatica, are ill-defined, overlapping, and often used incorrectly, contributing to the challenge of addressing LBP with existing clinical evidence [9]. The uncertainty that pervades LBP diagnosis and management also causes patients substantial distress [10].

To help distill the evidence for LBP and make it more widely available in clinical practice, one of the authors (RV) conducted prior work that defines an evidence-based examination for LBP in a chiropractic research setting [11]; systematically reviewed the literature of LBP diagnostic studies, which recommends standardizing diagnostic terminology for common conditions contributing to LBP [9]; and developed a revised office-based clinical examination [12] and paper-based clinical decision aid that coordinates working diagnoses with evidence-based nonpharmacological treatments offered by doctors of chiropractic [12,13].

To further the goal of supporting clinicians in applying the latest evidence for LBP in busy clinical settings, we leveraged this prior work to develop an electronic clinical decision support (CDS) tool. Our goal was to produce a web-based application to support evidence-based in-office examination, diagnosis, treatment decisions, and patient education for nonpathological conditions contributing to LBP. We used an iterative, user-centered design process to identify clinical requirements from published evidence, elicit user requirements from iterative interviews, and develop and evaluate the usability of an electronic CDS tool that facilitates and encourages evidence-based diagnosis and treatment.

## Methods

### Overview

**Figure 1.**
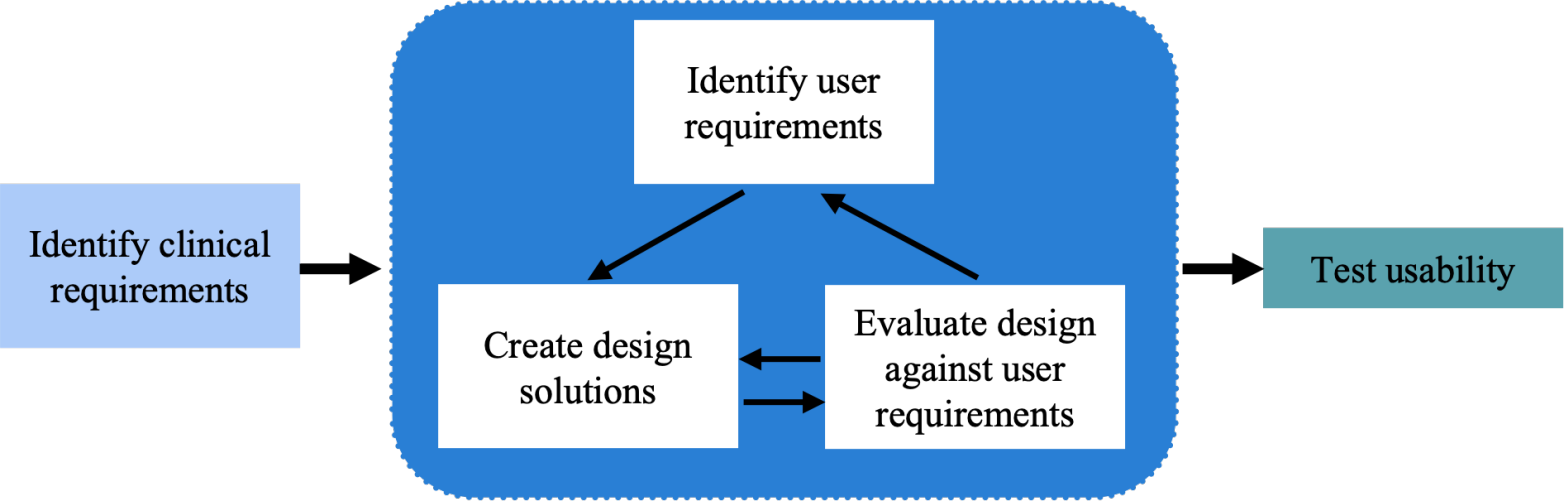
User-centered design process for low back pain decision support tool. After identifying relevant clinical findings from prior systematic literature reviews, we followed a standard, iterative process to identify and refine user requirements and design solutions before conducting a test of the usability using fictitious scenarios.

We conducted a three-step user-centered design process [14] to develop the CDS tool for LBP informed by best practices [15] in developing such tools: (1) identify clinical requirements based on our prior published reviews of the evidence for LBP diagnosis and treatment; (2) iteratively identify user requirements, create wireframes, and develop a working prototype; and (3) test usability as part of a preliminary assessment (Figure 1). Steps (1) and (2) occurred in June-August 2023, and Step (3) occurred in April and May 2024. We present the results of each step. User input was received during the second and third steps from 5 chiropractors serving clinical teaching faculty roles with Palmer College of Chiropractic. Participants were recruited from the College’s main campus in Davenport, Iowa (n=4) and branch campus in Port Orange, Florida (n=1). Each campus maintains teaching clinics staffed by licensed chiropractic doctors who provide care and oversee senior student clerkships. Faculty were recruited via an institutional review board (IRB)-approved email that offered information about the study, including participation requirements, and a request for a reply if interested. Those interested were sent additional information and scheduled for an interview.

Participants were targeted for recruitment as part of a purposive sampling strategy with the intention of including variation in terms of users by sex and experience. We intentionally targeted clinicians with more extensive experience in clinical practice and teaching, rather than novice users, to gain expert insights into usability and workflow issues in real-world settings and educational contexts. This approach helps ensure that the tool meets the demands and complexities of their working environments and builds credibility and acceptance among the clinical community. Users were recruited via email invitation; all invited users agreed to participate. Users verbally consented before each session. All user sessions were audio- and video-recorded.

### Clinical Requirements Identification

As described in the introduction, in prior work, we developed an evidence-based classification system for LBP, conducted a systematic review of LBP diagnostic studies, made recommendations for standardizing diagnostic terminology for common conditions contributing to LBP, and developed recommendations for LBP evaluation and treatment that were distilled in a series of publications including a paper-based clinical decision aid [9,11-13]. To identify clinical requirements for the CDS tool in this work, we reviewed our prior work to derive (1) clinical interview and examination inputs with adaptive characteristics that disable or enable items based on prior responses, (2) working diagnoses based on selected input items, and (3) evidence-based treatment recommendations corresponding to each working diagnosis. Furthermore, the clinician on the research team (RV) drafted patient education materials corresponding to each working diagnosis for user testing.

### User Requirement Elicitation and CDS Tool Design and Development

We iteratively elicited user requirements and developed and refined our prototype through wireframing and then software programming. We developed a discussion guide covering topics that included current approaches used for diagnosing and treating LBP, workflows related to LBP, pain points, ideas for tool design, and reactions to the wireframes showing the input items and example diagnoses, treatment recommendations, and patient educational information. Two research team members with expertise in informatics and design (RSR) and LBP clinical practice and research (RV) interviewed each user. Prior to each first design session, users were sent prior published literature related to LBP diagnosis and treatment. Users were asked to review in advance as a reminder of current evidence related to LBP evaluation, diagnosis, and treatment, and to gain familiarity with key sources underlying the CDS tool. Users were encouraged to “think aloud” during the design sessions.

After each design session, 1 research team member (RSR) reviewed the recording and summarized key findings using conventional content analysis [16] and the framework method [17]. Categories of findings were related to user requirements, changes were needed to make the wireframes and prototypes address the emerging requirements, and new design ideas emerged during the sessions organized by user task. Between design sessions, we brainstormed or ideated new design ideas for the tool and worked with an experienced user interface designer to create additional wireframes. We then developed a fully functional prototype of the tool as a web-based application. A second research team member (RV) reviewed the recordings and confirmed or amended key findings. Results were finalized through consensus among research team members (RSR and RV).

### Usability Test

To assess usability, 1 clinician-author (RV) developed 4 fictional clinical scenarios to help assess how the users would use the CDS tool in realistic situations. These involved a condition-specific history and examination findings for conditions matching criteria for acute nociceptive pain, chronic nociceptive pain, chronic nociceptive pain combined with symptoms of neurogenic claudication, and radicular pain. Each user was tested on 1 or 2 of these scenarios. At the start of each usability testing session, the user was given a brief overview of the tool and then asked to imagine that he or she was treating a patient with LBP. The user was then read a brief summary of a fictional patient’s LBP symptoms and was instructed to ask questions, as they would during a patient interview, to complete the input questions. The user was encouraged the “think aloud” as they used the CDS tool. After completing at least 1 scenario, the user was asked to complete the System Usability Scale (SUS), a 10-item measure that is commonly used to assess the usability [18]. SUS scores range from 0 to 100, with higher scores indicating greater usability. SUS scores >68 are considered above average, and those above 80 are in the top 10th percentile [19]. Users were encouraged to explain reasons for their answers and results were summarized [16].

### Ethical Considerations

This study was reviewed and approved by the RAND Human Subjects Protection Committee (HSPC ID: 2023-N0175). The IRB at Palmer College of Chiropractic was the relying IRB through a formal reliance agreement. All written and audiovisual data (which were not anonymized) were stored on secure servers accessible only to the research team. Because the interviews did not address protected or sensitive topics, but instead focused on perceptions of a potential website and clinical workflows, no written consent was obtained. Instead, verbal consent was obtained prior to conducting interviews. Participants were compensated US $150 per interview, with a maximum of 2 interviews.

## Results

### User Characteristics

All users practiced chiropractic in teaching clinics at Palmer College of Chiropractic at either an Iowa or Florida campus location. See Table 1 for user characteristics. All encountered LBP on a daily basis when they treated patients. Users varied on how often they attempted to arrive at a specific diagnosis from rarely to every visit. One user recently switched to an administrative position and was no longer engaging in direct patient care.

**Table 1.** Characteristics of chiropractors at Palmer College of Chiropractic (N=5) who participated in user design sessions to assess the low back pain decision support tool.

Characteristic	Statistical values
Sex, n (%)	
Male	2 (40)
Female	3 (60)
Years practicing chiropractic, mean (range)	18.5 (7-34)
Location, n (%)	
Iowa	4 (80)
Florida	1 (20)
Patient visits per week, n	~100
Years using an EHR^a^, mean (range)	10 (7‐12)

a EHR: electronic health record.

### Clinical Requirements

**Figure 2.**
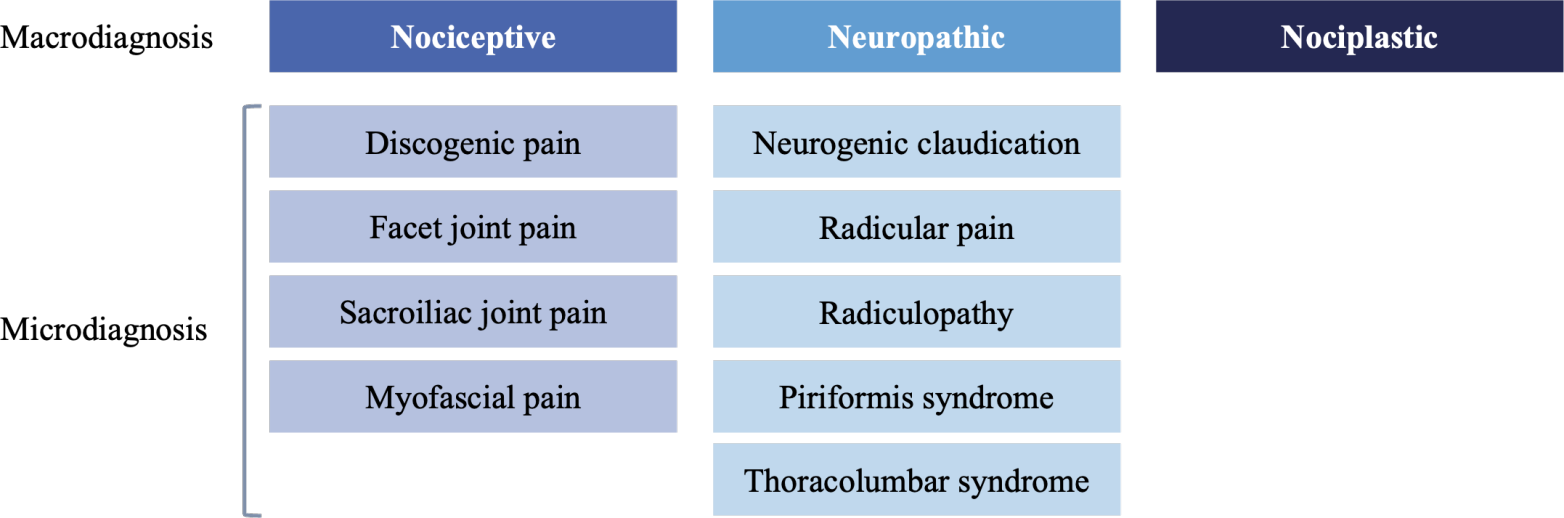
Types of low back pain. The literature identifies 3 macrodiagnoses and 9 microdiagnoses. These categories served as outputs of the decision support tool for low back pain.

We identified 14 possible working diagnoses divided into 3 macrocategories and 9 microcategories (Figure 2). For the input items and logic rules to enable them, we identified a total of 44 items: 13 interview items are enabled for all patients, 13 interview items are enabled conditional on other input items, 1 examination item is enabled for all patients, 16 examination items are enabled conditional on other input items, and 1 item is a synthesis of interview and examination findings and enabled for all patients. Items that were enabled for all patients helped identify macrodiagnoses; items that were conditionally enabled were related to microdiagnoses. For example, if the user selected “Sensory changes in a nerve root distribution,” which offers some evidence for the macrodiagnostic category of neuropathic pain, 8 interview items and 6 examination items are enabled to clarify 1 or more potential microdiagnoses.

To specify the clinical logic rules for each working diagnosis, we used Boolean logic. For example, piriformis syndrome was included as a possible diagnosis based on the following logic combining the relevant input items: “radiating pain into an ipsilateral leg” OR “tenderness of the greater sciatic notch” OR “buttock pain” OR “positive straight leg raise test” OR “increased pain with prolonged sitting.” The inputs and logic represent diagnostic information or criteria consistent with piriformis syndrome. If indicated by the literature, we included estimates of the strength of the evidence based on the quantity of inputs corresponding to a working diagnosis (eg, 1-5). Anchor terms of less likely (eg, 1) and more likely (eg, 5) are used because working diagnoses cannot be completely definitive.

Treatment recommendations were assigned to each working diagnosis based on published evidence. In many cases, 1 treatment recommendation applied to multiple diagnoses. For example, graded exposure and activity training are recommended for both neurogenic claudication and nociplastic pain. Some treatment recommendations apply to all working diagnoses (eg, education about condition and general exercise when safe and tolerated).

### User Requirements and CDS Tool Design

Example wireframes used in design sessions are shown in Figure S1 in Multimedia Appendix 1. Users were positive about the potential value of the CDS tool: “This tells you exactly where to focus your attention on. I like it” (User 3). Users said that the primary use case for using the tool would be when a new patient presents with LBP. Some might also use it if treatment was not working for reassessing their diagnoses and treatment plan and informing the potential for a referral. All users suggested that it would be useful both for practicing chiropractors and as an education tool.

User requirements and design features are shown in Table 2 organized by user task. User input led to key refinements in the design, such as organizing the input questions by microdiagnosis, adding a patient summary screen that persists during data input and when reviewing the output, adding more information buttons and graphics to input questions, and providing traceability by highlighting the input items used by the clinical logic to suggest a working diagnosis.

**Figure 3.**
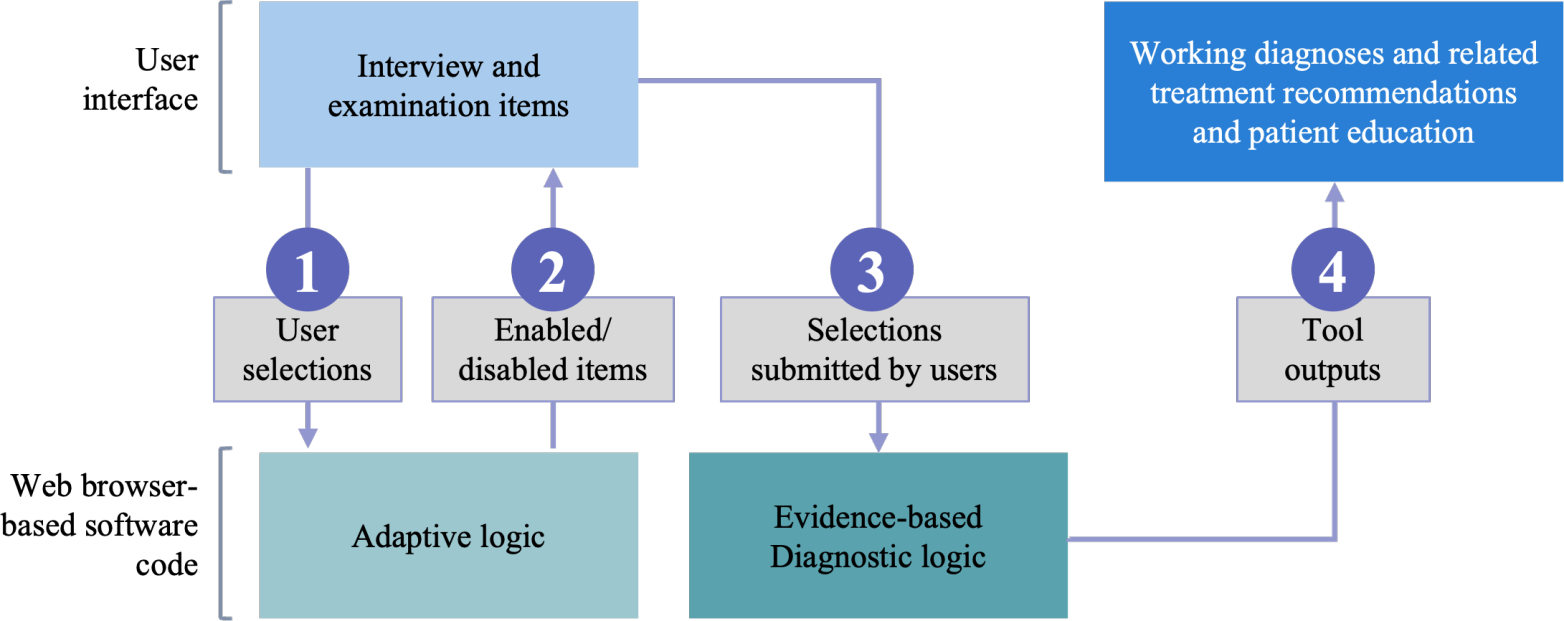
Technical architecture of web-based application for low back pain decision support. The sequence 1‐4 shows each step that occurs as the user interacts with the tool.

**Figure 4.**
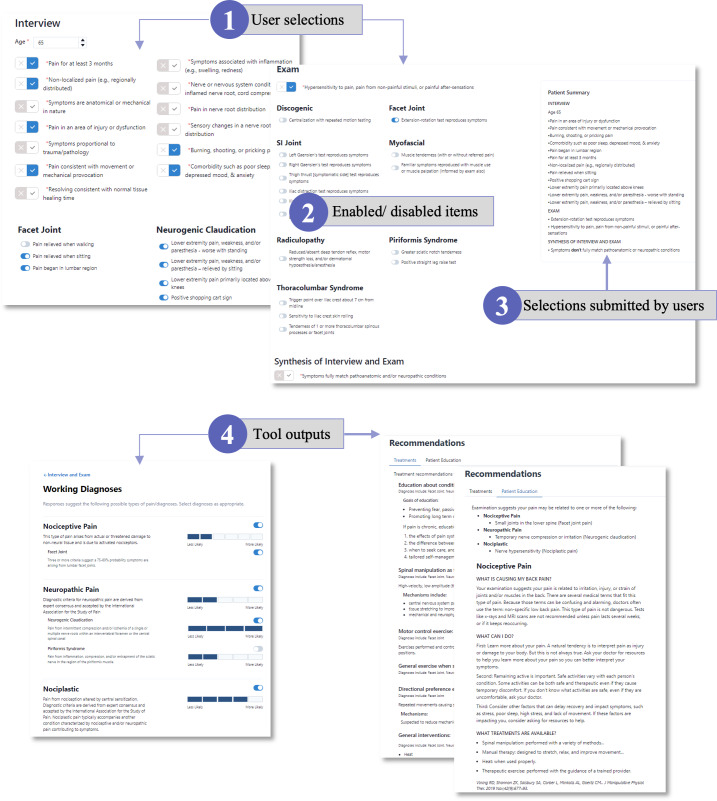
Complex multifactorial diagnosis with evidence for 3 macrodiagnoses of low back pain (nociceptive, neuropathic, and nociplastic pain). Screenshots of the tool show (1) user selections based on the interview and examination, with (2) items disabled according to the adaptive logic in the model. The greatest evidence suggests neurogenic claudication with overlying nociplastic pain. Piriformis syndrome was rejected by the model because symptoms overlap with neurogenic claudication and because historical and examination evidence is stronger for neurogenic claudication. The (3) summary panel to the right, shown only in the top right screenshot, shows the selections as submitted by the user. The bottom screenshots show the tool outputs (4), with the result of the evidence-based diagnostic logic displaying the working diagnoses (left) and recommendations for treatments and patient education (right).

The prototype CDS tool was built as a web-based application using the React javascript library and responsive design. For this prototype, all of the data entered by the user are stored within the user’s web browser—no database or server-side storage of data were used (Figure 3). Screenshots of the tool are shown in Figure 4 and Figures S2 and S3 in Multimedia Appendix 1.

**Table 2.** User requirements and design features by task for the low back pain decision support tool.

Task	User requirement	Design feature
Input interview and examination findings	Make it easy to input the relevant data.	Order interviews items before examination items to parallel clinical workflows; disable input items for microdiagnoses until they are indicated based on items for more general diagnoses; and cluster input items based on microdiagnosis.
	Allow the user to learn more about data that need to be inputted if desired.	Information buttons and graphics.
	Show a summary of the interview/examination findings as user enters them.	Patient summary section.
Review potential working diagnoses	Allow the user to see all potential working diagnoses, which reflects the multifactorial nature of LBP^a^, with relative strength of evidence based on interview/examination findings.	List diagnoses with likeliness scales to enable rapid visual confirmation of the extent of evidence supporting each condition, allowing users to confirm data accuracy and determine whether the interview and examination data align with the overall clinical presentation.
	For any specific working diagnosis, allow the user to view the specific interview/examination findings that constitute the evidence for it.	When user hovers over a diagnosis, highlight the related interview/examination findings in patient summary.
	Allow the user to go back and change/alter the interview/examination findings at any time to alter the potential working diagnoses.	Navigation arrow and button to return to the input screen.
Select potential working diagnoses	Allow the user to select/deselect from the list of working diagnoses based on the user’s clinical assessment.	Toggles for each diagnosis.
	Allow the user to document his or her selected working diagnoses in his or her notes.	Allow copy-paste; automated input into EHR^b^^,c^.
Save data for next visit	Allow the user to save his or her selected working diagnoses and interview/examination findings for review in future.	Save data tied to visit in EHR or database^b^.
Review treatment recommendations	Allow the user to see all evidence-based treatment recommendations for selected working diagnoses.	List treatments based on evidence-based guidelines for diagnoses.
	For any specific treatment recommendation, allow the user to see which diagnoses he or she corresponds to.	List diagnoses below each treatment recommendation.
Determine treatment recommendations	Allow the user to select/deselect and customizing treatment recommendations based on the user’s clinical assessment and experience with the various options.	Toggles for each treatment recommendation^b^.
Select relevant patient educational information	Allow the user to easily print the patient educational information.	Make educational information available in PDF or other printable format.
	Allow the user to select/deselect and adapt patient educational information.	Select buttons to turn on/off specific contents^b^.
	Allow the user to tailor the patient educational information based on patient and user preferences.	Let user edit content for each patient and configuration option for default content^b^.
Global	Allow the user to access the tool within the EHR.	Implement CDS^d^ tool within patient chart in EHR^b^.

a LBP: low back pain.

b Not yet implemented in prototype CDS tool.

c EHR: electronic health record.

d CDS: clinical decision support.

### Usability Test

The mean score of the SUS was 84.75 (range 67.5‐95). Qualitative results explained these findings (Table 3). Overall, users were enthusiastic about using the CDS tool because they perceived that it would help them focus on the most relevant information, especially for more complex patients. All users thought that the tool was easy to use from a technical perspective but required training and practice on the clinical content to use it effectively and efficiently. While using the tool during a study session, one user quickly transitioned from an initial impression that substantial effort would be needed to use it to expressing enthusiasm for the tool’s potential use for improving back pain diagnosis and treatment: “This (tool) is like walking into a storm, but it wouldn’t be like that the second time and certainly by the 50th time it would be fabulous” (User 4). One user said that the training needed to use the tool effectively was a good thing because that training would improve care for LBP—they strongly agreed with the statement from the SUS that said, “I needed to learn a lot of things before I could get going with this system” (User 3), but added that they meant that “in a positive connotation way.”

Users also reacted to the usability of specific features. When using the input page, user reactions included the following: enabling examination items only when indicated could help eliminate tests that might be done routinely but are not clinically relevant; the patient summary was useful for guiding the input process; the wording or clinical intention behind some items was difficult to understand (eg, “symptoms fully match pathoanatomic and/or neuropathic condition;” and “resolving consistent with normal tissue healing time”); and it might be difficult to have time to go through all items during a visit.

When using the list of working diagnoses, reactions from users included positive views of the feature that highlighted relevant input items when hovering over a diagnosis to show the inputs (ie, evidence) supporting it; clarity of the scale used to indicate strength of evidence; and helpfulness of having a list of diagnoses to prevent anchoring on one and also for explaining the clinical reasoning process to patients. Reactions to the treatment recommendations were generally positive and believed to be helpful for decision-making. Two users noted that they had preferences for some treatment options more than others based on what they were familiar with.

For the patient education materials, 3 users emphasized the importance of being able to print out copies. Two believed that the materials would be acceptable to patients while 3 found them too complex especially for those with low health literacy levels. Two users preferred handwriting education information to make it more personal. One preferred to print out the materials and highlight key information.

Users also described how they would likely use this CDS tool in their workflows. All users believed that it was important to make the tool available within the EHR to allow for easy access to it and to minimize the need to switch between applications or devices. All users would use the CDS tool while with the patients but 2 users mentioned that they might leave the room to use the tool separately. One suggested that a freestanding web-based version of the tool maybe useful as well in some scenarios. Two users wanted to modify their patient intake questions to match the CDS tool’s input items to help the clinician prepare for the visit and facilitate tool usage.

Users suggested several ideas to improve use and usability including use of colors on the input screen to break up the text more, autopopulating the CDS tool based on patient intake questions, providing a crosswalk between generated working diagnoses and *International Classification of Diseases, Tenth Revision* (*ICD-10*) codes to facilitate billing, indicating in the input screen if the combination input items selected did not make clinical sense and should be revisited, further emphasizing which treatments should be of higher or lower priority based on evidence (if it exists), and providing more visuals for patient education materials.

**Table 3.** Summary of qualitative usability evaluation results of the low back pain decision support tool.

Category	Selected finding	Illustrative quote
Overall impact of the CDS^a^ tool on clinical care	Users believed that the tool would help users focus on the relevant information, improve diagnostic accuracy, and use of evidence-based treatments.	“I’m very likely to use this… you know why? It makes me better. It makes me smarter… This is an elegant way to approach the black hole of low back pain.” (User 4) “It helps you focus your thoughts” (User 3)
Training required to use the CDS tool	Although the tool is simple to use technically, clinical knowledge is needed to use it effectively and will require training.	“It doesn’t seem complex; it just seems like I need to familiarize myself with it.” (User 1)
Input items	Enabling items adaptively can help guide information gathering.	“It is nice that it does close off those other options that maybe get in our way… I think this helps cater the treatment to the patient’s diagnoses rather than allowing the provider to cater the treatment to their own preferences.” (User 5)
	It might be challenging for users to go through all items during visits with a patient.	“I think it could be challenging even with familiarity… to answer it real-time [as] I’m having a conversation with somebody.” (User 1)
Working diagnoses	Degree of evidence is helpful and clearly shown in bar format.	“Very visually clear.” (User 4)
Treatment recommendations	The recommendations can help inform treatment for both clinician and student.	“Beyond nice, this is very educational… I love this, this is brilliant.” (User 3)
Patient education	The content may be helpful to some patients but too complex for others.	“I think this is very useful especially if someone starts catastrophizing.” (User 4) “I think it would be hugely beneficial at the report of findings to explain to the patient what the pain generator is and how that is affecting their presentation.” (User 5) “[For] 90% of [my patient] this would be far too complex… it is far beyond a lot of our patients understanding.” (User 3)

a CDS: clinical decision support.

## Discussion

### Principal Results

This study reports the initial development and evaluation of a CDS tool facilitating evidence-based diagnosis, clinical management, and patient education for LBP. The tool is web-based and can be embedded into the EHR for use during the clinical encounter as a support for interview, examination, care planning, and patient education. It can also be used independent of the patient encounter. The SUS score for the prototype was 84.75 (range: 67.5‐95), which is in the top 10th percentile. Users believed that the CDS tool was easy to use, although training in clinical content and practice was considered necessary for effective and efficient use. With such training and practice, users believed that it would improve care and shed light on the “black hole” of LBP diagnoses and treatment.

The CDS tool inputs included 44 patient interview and examination items that provided evidence of 12 possible working diagnoses. Input items were ordered to begin with interview items followed by examination items—conditionally displayed if relevant—so as to parallel clinical workflows. The output user interface allows multiple potential working diagnoses, reflecting the multifactorial nature of LBP. Likeliness scales enable rapid visual assessment of the extent of evidence supporting each condition. This feature enables clinicians to confirm data accuracy and determine whether the interview and examination data align with the overall clinical presentation. The ability to remove or reject working diagnoses for which there is little evidence in favor of others supported by stronger evidence can assist in prioritizing care. The patient education materials written at an eighth-grade level were designed to help further explain LBP, offer reassurance, list possible treatment and self-care options, describe other factors influencing pain severity (eg, stress, poor sleep, and lack of movement), and encourage patients to communicate with clinicians about these topics. Some interviews suggested that these materials had potential to be useful, although some revision may be needed. Review by, and feedback from, patients with LBP would best inform further development of this feature.

### Contribution and Comparison With Other Work

To our knowledge, this is the first provider-facing tool developed to assist clinicians in the diagnosis and treatment of LBP. A prior study describes the development a CDS tool to assist Australian pharmacists in advising patients about watchful waiting for acute LBP and recommending medications and provider types to seek for persistent symptoms [20]. Our work is complementary to that work in that it focuses on offering providers a CDS tool once patients seek care. The CDS tool offers a systematic process for a clinical evaluation which, in turn, generates working diagnoses and links these outputs with guideline-based nonpharmacological treatments offered by chiropractors and other practitioners providing similar treatments. Our tool also shares some design similarities with currently available web-based risk-screening tools, such as the Lung Cancer Screening Risk Calculator and the Fracture Risk Assessment Tool (FRAX) [21,22]. These calculators are clinician-facing applications with input variables (eg, age, sex, smoking status, and bone mineral density) for estimating the risk for future lung cancer and osteoporotic fracture.

In addition to distilling the evidence for LBP diagnosis and treatment and making it available to clinicians at the point of care, this tool has the potential to help clinicians address patient needs identified in qualitative research for LBP. Costa et al [10] describe difficulties encountered by people with LBP due to uncertainty stemming from diagnoses and treatment. Our CDS tool has the potential to support clinicians in explaining the nature of the problem, treatment plan, and what patients can do on their own to address the problem [10]. Cox et al [23] make specific recommendations to clinicians about communicating LBP diagnoses, including sharing thought processes and diagnostic reasoning and providing written information about diagnosis. Our tool addresses these recommendations [23].

The CDS tool we developed may enable clinicians to avoid common errors in clinical reasoning leading to misdiagnosis, such as anchoring and premature closure [24]. Anchoring is an attachment to a diagnosis despite contradictory evidence; premature closure occurs when a diagnosis is accepted before verification. By offering objective, evidence-based, and repeatable provisional working diagnoses based on history and examination data, with visual displays of the strength of evidence supporting working diagnoses, this tool may help clinicians more systematically and consciously consider each potential working diagnosis in the context of the strength of evidence.

### Limitations and Future Work

User input was gathered from 5 practicing chiropractic clinicians who participated in both the development and usability testing phases. This sample size is sufficient for formative research and for identifying major usability issues [25-27]. While there is a potential for bias due to their involvement in the design process, the likelihood of such bias is reduced, as the design sessions took place 7 months prior to the usability testing, limiting recall of specific design details. Informed by our findings and to address limitations in our scope, we plan to build on this work in 6 areas. First, we will implement the tool within a health system’s EHR to enable easy access within the clinical workflow and enhance certain features such as allowing users to save information for each patient. Second, we will iteratively refine the patient-facing content with patients, clinicians, and with consideration of evidence in literature. Third, we will expand to other clinical specialties that treat LBP. Our initial focus was on chiropractic, a profession focused on nonpharmacological diagnosis and treatment of neuro-musculoskeletal conditions and consisting of approximately 77,000 licensed practitioners in the United States [28]. There is potential to further develop the tool for physical therapy, primary care, and other specialties. Fourth, the tool may be expanded beyond the biological perspective to include other factors (eg, psychological, spiritual, environmental, and social) relevant to LBP diagnosis and management [29]. Fifth, we will examine opportunities to incorporate artificial intelligence (AI). AI-based approaches are gaining traction in diagnosis [30,31] and other areas. However, AI models are limited by the data quality used to create them. By standardizing the definition of interview and examination inputs, our tool has the potential to improve the quality of data used by AI. Finally, the CDS tool developed in this study does not screen for signs or symptoms of pathology (eg, fracture, infection, and malignancy). There is no current consensus on such screening, and findings can be interpreted differently with varied clinical histories and among patients in different age groups. However, inputs for major red flags (eg, history of cancer and pulsatile pain with abdominal bruit suggesting aortic aneurysm) can be added to prompt and facilitate the clinical screening process.

### Conclusions

The LBP tool we have developed has potential to help standardize the process of clinical evaluation leading to systematically derived working diagnoses that inform individually tailored treatment decisions. Our process of defining clinical requirements based on rigorous evidence reviews and applying user-centered design principles through eliciting user requirements produced a clinician-facing decision support tool that was viewed positively and with enthusiasm by clinical users. If implemented, this tool may shed light on the “black hole” of LBP care, facilitate dissemination of LBP evidence as it is produced, and catalyze innovation in LBP care.

## Supplementary material

10.2196/66666 Multimedia Appendix 1 Additional examples of wireframes and screenshots.

## Abbreviations

AI: artificial intelligence
CDS: clinical decision support
FRAX: Fracture Risk Assessment Tool
LBP: low back pain
SUS: System Usability Scale

## References

[1] Hartvigsen J, Hancock MJ, Kongsted A (2018). What low back pain is and why we need to pay attention. Lancet.

[2] Pitcher MH, Von Korff M, Bushnell MC, Porter L (2019). Prevalence and profile of high-impact chronic pain in the United States. J Pain.

[3] Dutmer AL, Schiphorst Preuper HR, Soer R (2019). Personal and societal impact of low back pain: the Groningen spine cohort. Spine (Phila Pa 1976).

[4] Wu A, March L, Zheng X (2020). Global low back pain prevalence and years lived with disability from 1990 to 2017: estimates from the Global Burden of Disease Study 2017. Ann Transl Med.

[5] Lo J, Chan L, Flynn S (2021). A systematic review of the incidence, prevalence, costs, and activity and work limitations of amputation, osteoarthritis, rheumatoid arthritis, back pain, multiple sclerosis, spinal cord injury, stroke, and traumatic brain injury in the United States: a 2019 update. Arch Phys Med Rehabil.

[6] Dieleman JL, Cao J, Chapin A (2020). US health care spending by payer and health condition, 1996-2016. JAMA.

[7] Maher C, Underwood M, Buchbinder R (2017). Non-specific low back pain. Lancet.

[8] Gräper PJ, Scafoglieri A, Clark JR, Hallegraeff JM (2024). Sensory profiles predict symptoms of central sensitization in low back pain: a predictive model research study. J Clin Med.

[9] Vining RD, Shannon ZK, Minkalis AL, Twist EJ (2019). Current evidence for diagnosis of common conditions causing low back pain: systematic review and standardized terminology recommendations. J Manipulative Physiol Ther.

[10] Costa N, Butler P, Dillon M (2023). “I felt uncertain about my whole future”-a qualitative investigation of people’s experiences of navigating uncertainty when seeking care for their low back pain. Pain.

[11] Vining R, Potocki E, Seidman M, Morgenthal AP (2013). An evidence-based diagnostic classification system for low back pain. J Can Chiropr Assoc.

[12] Vining RD, Minkalis AL, Shannon ZK, Twist EJ (2019). Development of an evidence-based practical diagnostic checklist and corresponding clinical exam for low back pain. J Manipulative Physiol Ther.

[13] Vining RD, Shannon ZK, Salsbury SA, Corber L, Minkalis AL, Goertz CM (2019). Development of a clinical decision aid for chiropractic management of common conditions causing low back pain in veterans: results of a consensus process. J Manipulative Physiol Ther.

[14] ISO (2019). ISO 9241-210:2019 —Ergonomics of human-system interaction — part 210: human-centred design for interactive systems. https://www.iso.org/standard/77520.html.

[15] Kawamoto K, Houlihan CA, Balas EA, Lobach DF (2005). Improving clinical practice using clinical decision support systems: a systematic review of trials to identify features critical to success. BMJ.

[16] Hsieh HF, Shannon SE (2005). Three approaches to qualitative content analysis. Qual Health Res.

[17] Gale NK, Heath G, Cameron E, Rashid S, Redwood S (2013). Using the framework method for the analysis of qualitative data in multi-disciplinary health research. BMC Med Res Methodol.

[18] Bangor A, Kortum PT, Miller JT (2008). An empirical evaluation of the System Usability Scale. Int J Hum Comput Interact.

[19] Measuring U (2011). Measuring usability with the System Usability Scale (SUS). https://measuringu.com/sus/.

[20] Downie AS, Hancock M, Abdel Shaheed C (2020). An electronic clinical decision support system for the management of low back pain in community pharmacy: development and mixed methods feasibility study. JMIR Med Inform.

[21] ScreenLC. Lung cancer screening risk calculator.

[22] FRAX Fracture risk assessment tool. https://frax.shef.ac.uk/FRAX/tool.aspx.

[23] Cox C, Hatfield T, Willars J, Fritz Z (2024). Identifying facilitators and inhibitors of shared understanding: an ethnography of diagnosis communication in acute medical settings. Health Expect.

[24] Rylander M, Guerrasio J (2016). Heuristic errors in clinical reasoning. Clin Teach.

[25] Nielsen Norman Group (2000). Why you only need to test with 5 users. https://www.nngroup.com/articles/why-you-only-need-to-test-with-5-users/.

[26] Virzi RA (1992). Refining the test phase of usability evaluation: how many subjects is enough?. Hum Factors.

[27] Shuldiner J, Kiran T, Agarwal P (2023). Developing an audit and feedback dashboard for family physicians: user-centered design process. JMIR Hum Factors.

[28] NBCE (2020). Practice analysis of chiropractic 2020—NBCE survey analysis. https://www.nbce.org/practice-analysis-of-chiropractic-2020/.

[29] King D (2015). Diagnosis.

[30] Fu Y, Zhou J, Li J (2024). Diagnostic performance of ultrasound-based artificial intelligence for predicting key molecular markers in breast cancer: a systematic review and meta-analysis. PLoS ONE.

[31] Nowroozi A, Salehi MA, Shobeiri P (2024). Artificial intelligence diagnostic accuracy in fracture detection from plain radiographs and comparing it with clinicians: a systematic review and meta-analysis. Clin Radiol.

