# Supplementary material for: Addressing the “Black Hole” of Low Back Pain Care With Clinical Decision Support: User-Centered Design and Initial Usability Study
Source: JMIR Form Res. 2025 Feb 4;9:e66666. doi: 10.2196/66666 (PMC11813196; doi:10.2196/66666)

**Appendix: Additional examples of wireframes and screenshots.**

**Figure S1: Initial wireframe used in user-centered design process for low back pain clinical decision support tool.**


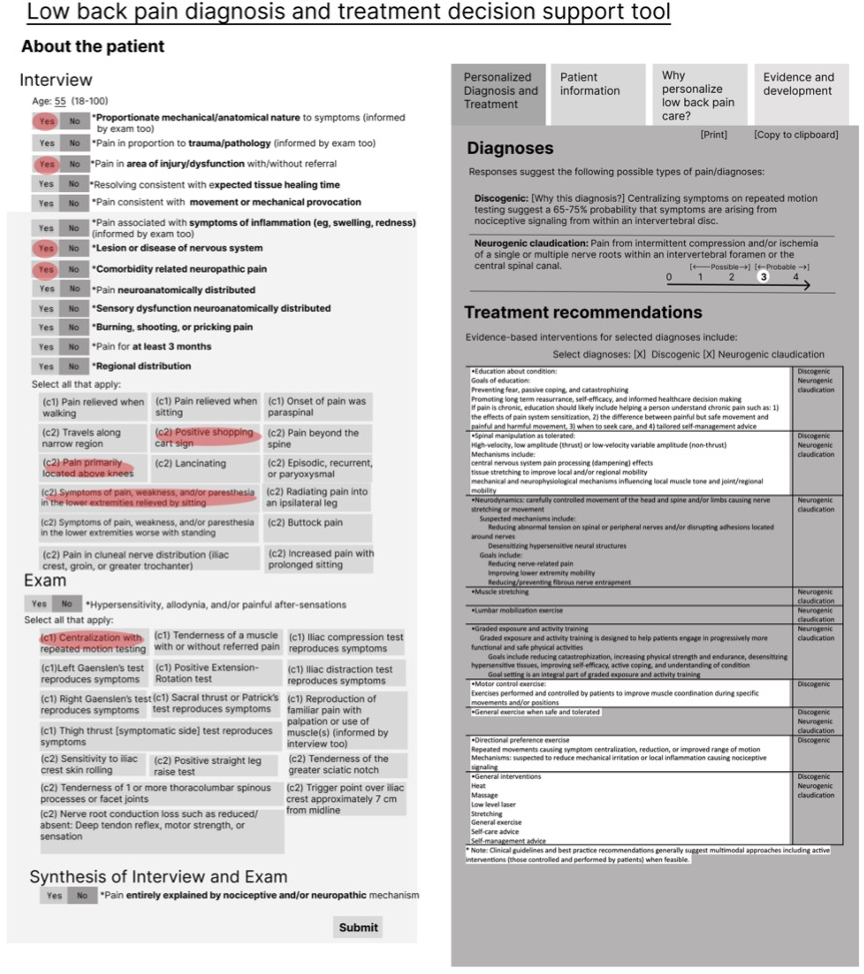


**Figure S2: Additional example screenshots of low back pain clinical decision support tool for complex diagnosis involving Nociceptive + Neuropathic pain: Discogenic pain, radicular pain, and radiculopathy.**


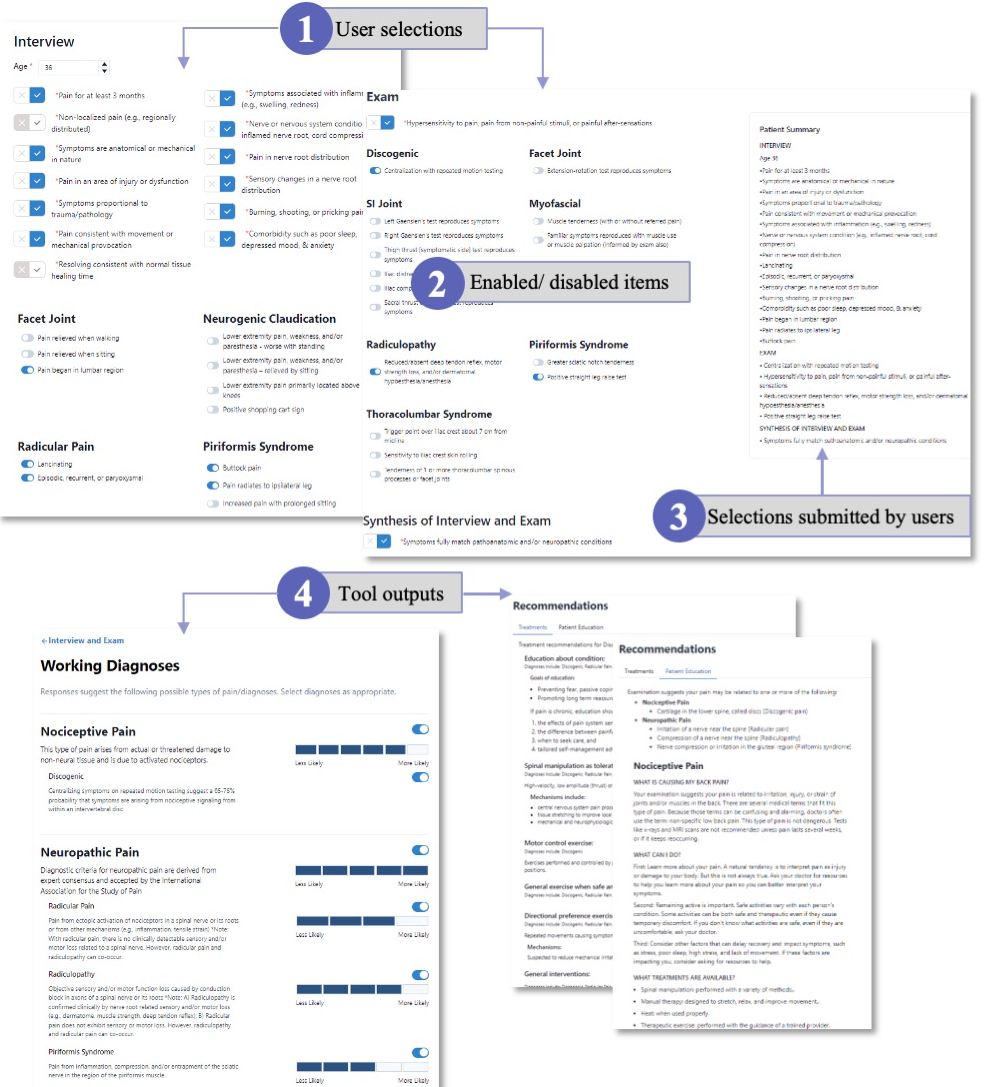


**Figure S3: Additional example screenshots of low back pain clinical decision support tool for less complex diagnosis suggesting Nociceptive pain most likely arising from Discogenic and facet sources**


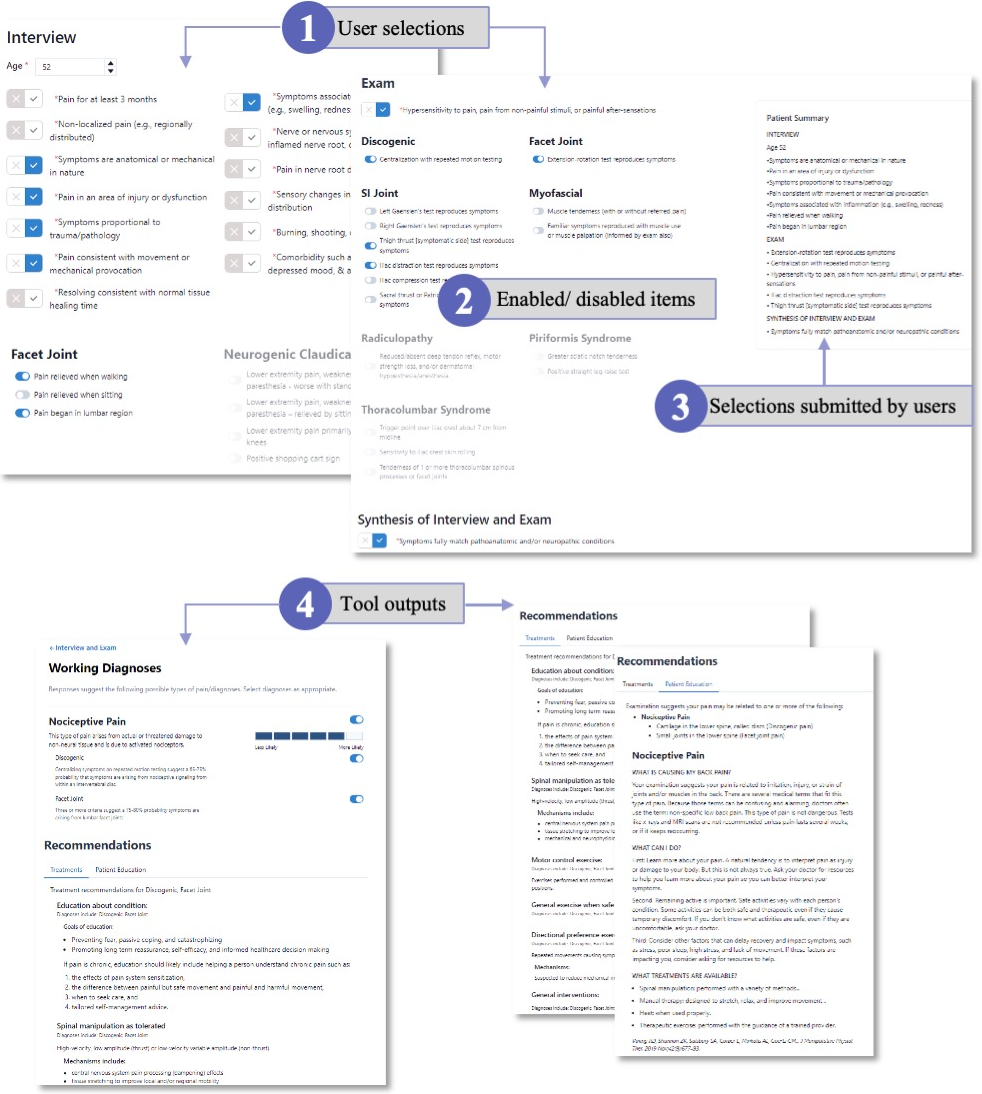

Supplement: Multimedia Appendix 1 [file formative-v9-e66666-s001.docx]
